# RNA-Based Detection Does not Accurately Enumerate Living *Escherichia coli* O157:H7 Cells on Plants

**DOI:** 10.3389/fmicb.2016.00223

**Published:** 2016-02-26

**Authors:** Wenting Ju, Anne-Laure Moyne, Maria L. Marco

**Affiliations:** ^1^Department of Food Science and Technology, University of California, Davis, DavisCA, USA; ^2^Western Center for Food Safety, University of California, Davis, DavisCA, USA

**Keywords:** phyllosphere, foodborne pathogen, EHEC, RT-qPCR, PMA-qPCR, detection, fresh produce, viability

## Abstract

The capacity to distinguish between living and dead cells is an important, but often unrealized, attribute of rapid detection methods for foodborne pathogens. In this study, the numbers of enterohemorrhagic *Escherichia coli* O157:H7 after inoculation onto Romaine lettuce plants and on plastic (abiotic) surfaces were measured over time by culturing, and quantitative PCR (qPCR), propidium monoazide (PMA)-qPCR, and reverse transcriptase (RT)-qPCR targeting *E. coli* O157:H7 *gapA*, *rfbE*, *eae*, and *lpfA* genes and gene transcripts. On Romaine lettuce plants incubated at low relative humidity, *E. coli* O157:H7 cell numbers declined 10^7^-fold within 96 h according to culture-based assessments. In contrast, there were no reductions in *E. coli* levels according to qPCR and only 100- and 1000-fold lower numbers per leaf by RT-qPCR and PMA-qPCR, respectively. Similar results were obtained upon exposure of *E. coli* O157:H7 to desiccation conditions on a sterile plastic surface. Subsequent investigation of mixtures of living and dead *E. coli* O157:H7 cells strongly indicated that PMA-qPCR detection was subject to false-positive enumerations of viable targets when in the presence of 100-fold higher numbers of dead cells. RT-qPCR measurements of killed *E. coli* O157:H7 as well as for RNaseA-treated *E. coli* RNA confirmed that transcripts from dead cells and highly degraded RNA were also amplified by RT-qPCR. These findings show that neither PMA-qPCR nor RT-qPCR provide accurate estimates of bacterial viability in environments where growth and survival is limited.

## Introduction

Enterohemorrhagic *Escherichia coli* serotype O157:H7 is an organism that causes diseases ranging from self-limiting diarrhea to life-threatening hemolytic uremic syndrome (HUS) and is one of the most important foodborne pathogens worldwide (Pennington, 2010). According to the United States Centers for Disease Control and Prevention, *E. coli* O157:H7 causes 63,153 cases of infection per year in the US alone (Scallan et al., 2011). In recent years, leafy greens have been increasingly associated with *E. coli* O157:H7 outbreaks (Doyle and Erickson, 2008). Although *E. coli* O157:H7 does not survive well or grow to high numbers on intact plants in the field, very low numbers of viable cells are potential sources of infection (Tuttle et al., 1999). Thus, sensitive and specific *E. coli* O157:H7 detection methods are required to effectively prevent foodborne outbreaks and sporadic infections resulting from consumption of fresh produce.

Traditional culture methods for detection of *E. coli* O157:H7 employ enrichment followed by isolation on selective and differential media such as sorbitol-MacConkey or CHROMagar O157 (Feng et al., 2011). Culture-based approaches are expensive, lab intensive, and time consuming, and might potentially result in false negatives due to the presence of viable cells unable to form colonies on standard laboratory culture media. Alternatively, culture-independent molecular detection methods such as PCR can significantly reduce detection times and increase specificity. PCR, in particular, is a widely applied method that is increasingly used for detection of foodborne pathogens. However, a shortcoming of this method is that DNA is typically not rapidly degraded in intact cells and therefore standard PCR and quantitative PCR approaches are not able to distinguish between living and dead bacteria (Lauri and Mariani, 2009). One approach used to discriminate between viable and dead cells is the inclusion of propidium monoazide (PMA) prior to DNA extraction and PCR amplification of pathogen-specific target genes (Nocker et al., 2007). PMA penetrates into bacteria with compromised cell membranes and binds genomic DNA (Nocker et al., 2007). Exposure of the bacteria to light activates the azide group resulting in DNA modification and renders the DNA recalcitrant to PCR amplification. However, the degree of membrane permeability can vary and high numbers of dead cells may interfere with PMA-PCR quantification of the viable cell fraction, as demonstrated by our recent work as well as by others (Li and Chen, 2013; Moyne et al., 2013; Pacholewicz et al., 2013; Barbau-Piednoir et al., 2014).

Bacterial transcripts are regarded to have short half-lives and high turn-over rates (Richards et al., 2008). This property has been crucial to the development of targeted and global gene expression analyses as a means to identify and compare physiologically relevant cellular responses. Another application is to use mRNA as indicator for viability. It has been assumed that because bacterial transcripts are sensitive to degradation by intra- and extra-cellular RNases, mRNA levels should rapidly decline after death. Therefore, unlike DNA-based detection, mRNA would only be limited to the viable and active cells within the population. Based on this assumption, reverse-transcriptase quantitative PCR (RT-qPCR) assays have been developed to detect a variety of foodborne pathogens such as enterohemorrhagic *E. coli*, *Listeria monocytogenes, Salmonella*, *Vibrio*, and *Campylobacter* species (Klein and Juneja, 1997; Sheridan et al., 1998; Szabo and Mackey, 1999; McIngvale et al., 2002; Yaron and Matthews, 2002; de Wet et al., 2008; D’Souza et al., 2009; Miller et al., 2010; Techathuvanan et al., 2010; Kurakawa et al., 2012; Zhou et al., 2014). However, while some reports have concluded that mRNA disappears quickly after cell death (McIngvale et al., 2002), other findings suggest that transcripts can persist for extended lengths of time (e.g., Sung et al., 2005; Xiao et al., 2012). Therefore, detailed investigations of transcript-based methods to enumerate viable bacterial cell numbers are urgently needed.

The aim of this study was to apply and compare RT-qPCR, PMA-qPCR, qPCR and culturing methods for detection of viable *E. coli* O157:H7. These methods were investigated for the pathogen on lettuce, following exposure to desiccation conditions on a sterile plastic surface, after different cell inactivation treatments, and for purified RNA exposed directly to ribonuclease.

## Materials and Methods

### Bacterial Strain and Lettuce Growth Conditions

Rifampicin-resistant *E. coli* serotype O157:H7 strain ATCC 700728 (lacking *stx1* and *stx2*) was used in previous field trials (Moyne et al., 2013; Williams et al., 2013). The strain was routinely grown in Tryptic Soy Agar (TSA) or Tryptic Soy Broth (TSB) under agitation (250 rpm) at 37°C. Cells were inactivated by incubation in 70% isopropanol or 92°C for 10 min, and death was confirmed by examining for growth on TSA plates incubated at 37°C for at least 48 h. For selective growth of ATCC 700728, rifampicin (Rif; Gold Biotechnology, St. Louis, MO, USA) was added to the TSA at a final concentration of 50 μg/ml.

Seeds of Romaine lettuce (*Lactuca sativa*) cv. Braveheart were grown in Sunshine mix potting soil (Sun Gro Horticulture Distribution, Bellevue, WA, USA) with a light density of 230 μmol m^-2^ s^-2^ and 60% relative humidity (RH) in an environmental chamber (PGR15, Conviron, Pembina, ND). Day (12 h) and night time (12 h) temperatures were set at 23 and 18°C, respectively. One day before *E. coli* inoculation, 4-week old lettuce plants were moved to a growth chamber in the lab (Percival, Geneva Scientific LLC, Fontana, WI, USA) maintained as described above except with a RH of 30%.

### Measurements of *E. coli* O157:H7 on Lettuce

Living or dead (killed by incubation in 70% isopropanol for 10 min) exponential-phase *E. coli* ATCC700728 cells were collected by centrifugation at 5,000 *g* for 3 min at 22°C, and the cells were washed twice with phosphate buffered saline (PBS; 137 mM NaCl, 2.7 mM KCl, 10 mM Na_2_HPO_4_, and 2 mM KH_2_PO_4_, at pH 7.0). Romaine lettuce plants were inoculated with 20 10-μl aliquots of either the viable or inactivated *E. coli* ATCC 700728 cell suspensions to result in approximately 10^8^ cells per leaf and then immediately returned to the growth chamber maintained at 30% RH. Leaves were sampled prior to *E. coli* inoculation and then at 0, 3, 24, 48, 72, and 98 h. Each sample consisted of two lettuce leaves that were randomly excised at the stem with sterile forceps and immediately transferred into a Whirl-pak bag (Nasco, Fort Atkinson, WI, USA) containing 100 ml sterile 0.1% peptone (Becton Dickinson, Franklin lanes, NJ, USA). Bacterial cells were dislodged from the lettuce by sonication at room temperature for 7 min in a Branson 8510 Ultrasonicator (Branson Ultrasonics Corporation, Danbury, CT, USA). For *E. coli* ATCC700728 colony enumeration, serial dilutions of lettuce washes were plated onto TSA containing 50 μg/ml rifampicin using an automated spiral plater (Autoplate 4000, Spiral Biotech Inc., Boston, MA, USA) and incubated at 37°C for at least 24 h. The same lettuce leaf washes were also used for DNA extraction with and without PMA exposure. For RNA isolation, two lettuce leaves were transferred to a Whirl-pak bag containing 100 ml ice-cold, acidic-phenol, ethanol RNA-stabilizing solution [5 mL water-saturated phenol (pH 6.6), 95 mL ethanol, 0.9 L RNase free water] (Yu et al., 2013), and cells were dislodged by ultrasonication 7 min in an ice cold water bath. For DNA and RNA extractions, 40 ml of the suspensions were centrifuged at 15,000 *g* for 3 min at 4°C. The cell pellets were washed twice with Tris-EDTA (TE, 10 mM Tris, 1 mM EDTA, pH 7.5). Fractions designated for PMA were then treated as described below, and the remaining samples were flash frozen in liquid nitrogen, and then stored at -80°C.

### Measurements of *E. coli* on Abiotic Surfaces

Living or dead (killed by incubation in 70% isopropanol for 10 min) exponential-phase *E. coli* ATCC700728 were collected by centrifugation at 5,000 *g* for 3 min at 22°C, and the cells were washed twice with PBS as described above. The *E. coli* suspensions were inoculated in 20 10-μl aliquots onto sterile petri dishes (Fisher Scientific, Pittsburgh, PA, USA) to reach approximately 10^8^ cells and the petri dishes were left open in a biosafety cabinet (Baker Company, Sanford, ME, USA) at the ambient RH of approximately 30%. At multiple times, petri dishes were collected and the *E. coli* cells washed off using 1 ml of 0.1% peptone for colony enumeration or genomic DNA extraction. For RNA extraction, *E. coli* cells were suspended directly from the petri dishes with ice-cold, acidic-phenol, ethanol RNA-stabilizing solution as described above. For nucleic acid extractions, the cell suspensions were centrifuged at 12,000 *g* for 2 min at 4°C, the pellet was washed twice with TE buffer and either exposed to PMA (see below) or immediately flash frozen in liquid nitrogen and stored in -80°C.

### PMA Treatment and DNA Extraction

A fraction of the cells recovered from Romaine lettuce or petri dishes were exposed to PMA (Biotum, Hayward, CA, USA) as described previously (Moyne et al., 2013). Briefly, cell suspensions were incubated in the dark for 30 min with shaking (500 rpm) in the presence of a final concentration 0.04 mM PMA. The cells were placed on ice and then exposed to a 500 W halogen light at a distance of 20 cm for 3 min prior to DNA extraction. For testing the interference of dead cells in PMA-qPCR detection, 10^6^ exponential-phase *E. coli* ATCC700728 cells inactivated in 70% isopropanol were mixed with different proportions of viable, exponential-phase cells of that strain in 0.1% peptone water prior to PMA exposure. For DNA extraction, bacteria (with or without exposure to PMA) were lysed by boiling for 5 min in Prepman solution (Life Technologies, Foster City, CA, USA) and debris was removed by centrifugation at 10,000 *g* for 2 min.

### RNA Extraction and Digestion

Bacterial cells were lysed by incubation in 200 μl TE (30 mM Tris⋅Cl, 1 mM EDTA), containing 0.5 mg proteinase K (Qiagen, Valencia, CA, USA) and 15 mg/ml lysosome (Sigma–Aldrich, St Louis, MO, USA) for 30 min at 25°C. Total RNA was then purified with the RNeasy mini kit according to the manufacturer’s instructions (Qiagen, Valencia, CA, USA). Remaining DNA was digested with TURBO DNase (Life Technologies) prior to measuring RNA quality on the Agilent 2100 Bioanalyzer system with the Agilent RNA 6000 Nano kit (Agilent, Santa Clara, CA, USA).

RNA digests were performed by incubating 400 ng high-quality (RIN:2.0) *E. coli* ATCC700728 RNA in 0.1 ng of RNase A (0.1 μg/ml; Life Technologies) and nuclease-free water (Life Technologies) at 37°C for different lengths of time. To inhibit RNase activity, 40 U of RNase inhibitor (Life Technologies) was added at the indicated time points.

### Reverse Transcriptase (RT)-qPCR

Reverse transcription was performed using RETROscript^®^ Reverse Transcriptase as indicated by the RETROscript kit with included random decamers (Life Technologies). To confirm the absence of genomic DNA, negative control reactions contained RNA and all reagents except for the reverse transcriptase (no-RT controls).

To quantify DNA and cDNA, qPCR was performed on an ABI 7500 Fast Real-time PCR system (Life Technologies) using 0.20 μM forward and reverse primers (**Table 1**), 1X Ssofast EvaGreen Supermix (Bio-Rad, Hercules, CA, USA), and 2 μl gDNA or cDNA template. Cycling conditions included an initial activation step at 95°C for 10 s, followed by 40 cycles of denaturation at 95°C for 5 s and annealing/extension temperatures at 60°C for 30 s. Melting curves were then performed by increasing the temperature from 60 to 95°C at 0.2°C/10 s and recording the fluorescence. Threshold cycle (*C*_t_) values were automatically generated by the 7500 Fast Real-Time PCR software. All (RT)-qPCR assays were performed in triplicate. PCR amplification efficiency for each of the primer pairs was similar to the values reported previously (Sharma, 2006; Sharma et al., 2010) and at 91% for the gapA primers developed here. Negative controls for PCR and RT-qPCR consisted of reactions lacking template DNA. No-RT controls were also used for all RT-qPCR assays and were confirmed to either lack any detectable amplification (*C*_t_ over 40) or exhibited a *C*_t_ that was at least five cycles higher than the sample cDNA template. To quantify the number of *E. coli* ATCC 700728 cells, standard curves were included for each (RT)-qPCR run by amplifying a 5 log_10_ dilution of either gDNA or cDNA (ratio of 23S rRNA to 16S rRNA = 1.8) from a known number of viable, exponential-phase *E. coli* ATCC 700728 cells. For all genes, the lower limits of detection by qPCR were between 100 and 1000 cells and for RT-qPCR between 200 and 500 cells.

**Table 1:** Primer sets used for qPCR, PMA-qPCR, and RT-qPCR.

Target	Primer sequence	Size (bp)	Reference
lpfA	5′- CACCGTTAAGAGCGACCAGGG -3′	165	Sharma et al., 2010
	5′- GAAGATTGCGATACCACCACG -3′		
eae	5′- TCTGTGTGGATGGTAATAAATTTTTG -3′	105	Sharma, 2006
	5′- GTAAGTTACACTATAAAAGCACCGTCG -3′		
rfbE	5′- TCAAAAGGAAACTATATTCAGAAGTTTGA -3′	129	Sharma, 2006
	5′- CGATATACCTAACGCTAACAAAGCTAA-3′		
gapA	5′-TAGGGGGCAGATTTTATATTCCGT-3′	143	This study
	5′-AACCAATGCTCCTATCACACCAAT-3		


### Statistical Analysis

Microbial data [colony forming units (CFU) or estimated cells per leaf or plate] were log transformed before examining with JMP Pro 12 software (SAS Institute Inc., Cary, NC, USA). Because the data did not follow a normal distribution, the nonparametric Kruskal–Wallis test was used with a 5% significance level. If a significant effect was found, pair-wise comparisons were performed using the Steel-Dwass test. For comparing the different methods used to estimate the number of live and dead cells, data obtained from all amplicons and time points were combined. For comparing the four amplicons used to quantify the cell numbers, data for each time point were combined according to the method of quantification.

## Results

### Comparison of Transcript-Based Enumeration of *E. coli* O157:H7 on Romaine Lettuce to Culture and DNA-Based Methods

**FIGURE 1:**
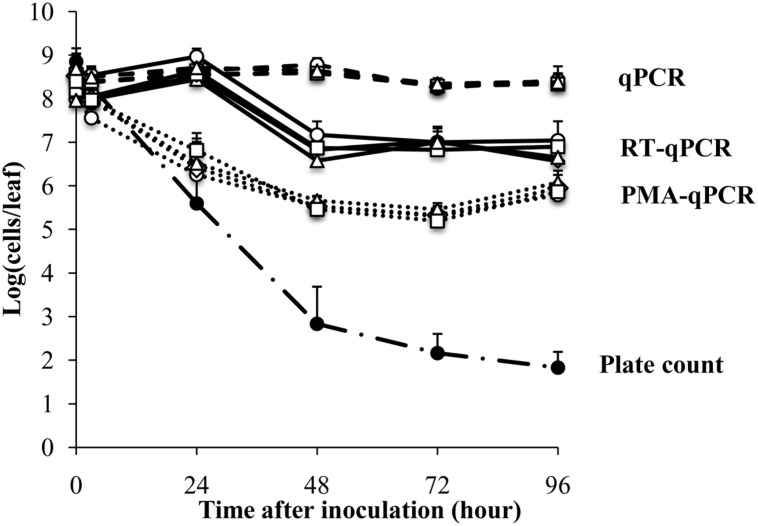
**Survival of *Escherichia coli* ATCC 700728 on Romaine lettuce leaves in the growth chamber at 30% RH.** *E. coli* O157:H7 ATCC 700728 abundances were estimated by plate counts (CFU on TSA containing Rif), qPCR, PMA-qPCR and RT-qPCR. *gapA* (♢), *eae* (□), *lpfA* (△), and *rfbE* (○) were used to estimate the *E. coli* cell numbers by qPCR, PMA-qPCR, and RT-qPCR. Each point represents the mean ± standard deviation of three independent replicates.

*Escherichia coli* O157:H7 strain ATCC700728 was inoculated onto 4-week old Romaine lettuce plants in small aliquots (10 μl) to avoid aerosolization as previously described (Moyne et al., 2013). The plants were then incubated at 30% RH and near ambient temperatures (18 and 23°C) in an environmental chamber. Estimates of viable cells according to growth on TSA showed that the numbers of *E. coli* ATCC 700728 decreased sharply after application onto the plants from 8.8 log_10_ colony forming units (CFU) per leaf to 2.8 log_10_ CFU per leaf within the first 48 h (**Figure 1**). By 96 h, culturable *E. coli* decreased to only 1.8 log_10_ CFU per leaf (**Figure 1**).

In contrast to culture-based estimates, qPCR enumeration of *E. coli* ATCC 700728 showed that the total number of (living and dead) *E. coli* cells on the Romaine lettuce did not change from inoculum levels (**Figure 1**). These findings did not differ between any of the housekeeping [*gapA* (glyceraldehyde-3-phosphase dehydrogenase)] and virulence [*eae* (intimin), *lpfE* (long polar fimbriae), and *rfbE* (O-antigen transporter)] genes tested and estimates of total cell numbers were similar for all target genes at all time points (Kruskal–Wallis, *P* = 0.5502). To attempt to quantify only the viable fraction of *E. coli* cells on lettuce, plant leaf washes were also incubated in PMA prior to DNA extraction and qPCR. One day (24 h) after *E. coli* inoculation, cell quantities measured by PMA-qPCR were similar to those estimated by culturing on laboratory culture medium (Steel-Dwass, *P* = 0.7838) and significantly lower than estimates according to qPCR alone (Steel-Dwass, *P* = 0.0002; **Figure 1**). However, when measured again 1 day later and at all subsequent time points, the three methods yielded different results. Notably, PMA-qPCR indicated only a 10-fold decline in *E. coli* cell numbers whereas culture-based estimates were 1000-fold lower and these differences did not change for the remainder of the 4 days experiment.

Lastly, transcript quantification was used as an approach to estimate viable *E. coli* O157:H7 on lettuce. Transcript levels for each of the four target genes, *gapA, eae*, *lpfE*, and *rfbE*, did not change according to RT-qPCR from inoculum levels during the first 24 h after inoculation of *E. coli* ATCC 700728 into Romaine lettuce (**Figure 1**). To this regard, there were no significant differences between the numbers of cells estimated by RT-qPCR and qPCR at that time (Steel-Dwass, *p* = 0.9998). One day later, RT-qPCR suggested that *E. coli* cell numbers were reduced by 100-fold to approximately 10^7^ cells per leaf; a value significantly lower than detected according to qPCR (Steel-Dwass, *P* = 0.0002). Estimates of cell quantities did not decline further for the remainder of the study. This finding was not likely due to differences in gene expression because *E. coli* cell enumerations were equal for all four target genes tested (Kruskal–Wallis, *P* = 0.4869; **Figure 1**). Because the ratio of 23S rRNA to 16S rRNA is a proxy for RNA integrity in bacterial cells (Sambrook et al., 1989), we also measured these ratios for the total RNA recovered from lettuce. Unlike the high-quality RNA extracted from the *E. coli* inoculum (23S rRNA to 16S rRNA ratio of 1.8), the ratio was at 1.1 ± 0.4 for RNA recovered from the lettuce 3 h after *E. coli* ATCC 700728 inoculation (**Supplementary Figure S1**). This ratio was reduced to 0.8 ± 0.3 at each sampling point for the following 3 days and declined further to 0.3 ± 0.2 by the last day of the study (**Supplementary Figure S1**).

### Survival of *E. coli* ATCC700728 on a Sterile Plastic Surface

**FIGURE 2:**
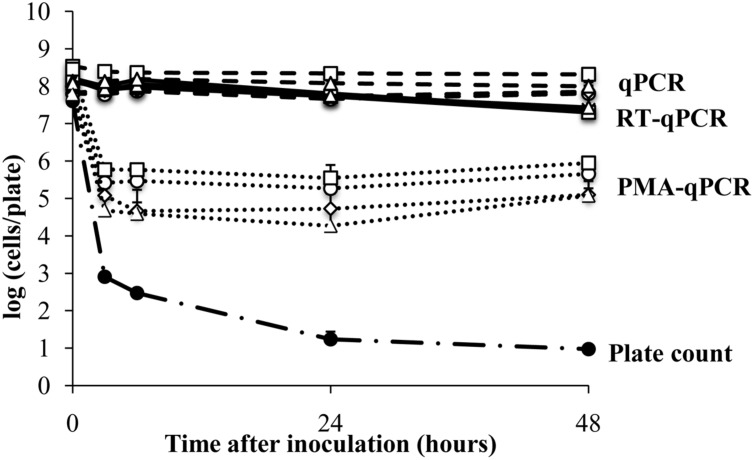
**Survival of *E. coli* ATCC 700728 on a plastic surface.** *E. coli* ATCC 700728 cell abundances were estimated by plate counts (CFU on TSA containing Rif), qPCR, PMA-qPCR and RT-qPCR. *gapA* (♢), *eae* (□), *lpfA* (△), and *rfbE* (○) were used to estimate the *E. coli* cell numbers by qPCR, PMA-qPCR and RT-qPCR. Each point represents the mean ± standard deviation of three independent replicates.

The plant surface contains nutrients and protected sites that might induce gene expression or support the viability of bacterial colonists, even in the absence of growth. Therefore, we next examined *E. coli* ATCC700728 survival on an abiotic surface (sterile petri dishes) to measure whether such *in planta*-variation could have resulted in differences in viable *E. coli* cell estimates. According to CFU enumeration on TSA, the number of living *E. coli* ATCC 700728 declined by 4.7 logs (7.6 log_10_ CFU/leaf to log 2.9 log_10_ CFU per plate) within 3 h after application onto the plastic surface (**Figure 2**). After 48 h, only an average of 10 CFU per plate was detected. In contrast, total *E. coli* ATCC700728 cells according to qPCR for all genes examined did not change and remained at inoculum levels (**Figure 2**). PMA-qPCR estimates showed a 100-fold decrease in viable *E. coli* cells within the first 3 h after inoculation, but then cell numbers were constant and remained at approximately 5.5 log_10_ cells per plate at subsequent time points (**Figure 2**). By comparison, cell numbers estimated by RT-qPCR for all four target genes declined less than 10-fold compared with the inoculum and were not significantly different from those estimated by qPCR (Kruskal–Wallis, *p* = 0.0656; **Figure 2**). Shortly (3 h) after inoculation onto the abiotic surface, the integrity of the RNA was high according to 23S and 16S rRNA ratios (1.8). These ratios then declined 24 h later to 1.5 ± 0.2 and remained at those levels until the end of the study (**Supplementary Figure S2**).

### Detection Limit for Viable Cells by PMA-qPCR

**FIGURE 3:**
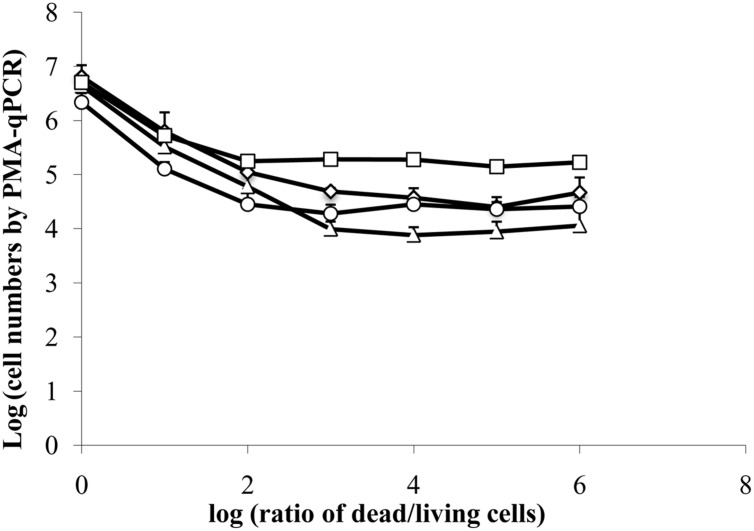
**Interference of dead cells in the detection of viable cells by PMA-qPCR.** A total of 6.7 log_10_ isopropanol-treated (dead) *E. coli* ATCC 700728 cells were mixed with 0.7 to 6.7 log_10_ exponential-phase *E. coli* ATCC 700728 in 10-fold cell increments. The cell mixtures were then exposed to PMA followed by DNA extraction and qPCR. Cell numbers were estimated by targeting *gapA* (♢), *eae* (□), *lpfA* (△) and *rfbE* (○) genes compared to standard curves constructed using known quantities of *E. coli* cells. Each point represents the mean ± standard deviation of three independent replicates.

Because estimates of viable cells according to PMA-qPCR were significantly higher than those detected by CFU enumeration starting 48 h after inoculation of *E. coli* ATCC 700728 onto lettuce, we investigated whether a high number of dead cells, such as the 10^6^ fold reduction in living cells estimated by culturing, might interfere with the accuracy of PMA-qPCR. To test this possibility, we mixed different proportions of isopropanol-treated (dead) *E. coli* ATCC700728 cells with exponential-phase *E. coli* ATCC700728 in 10-fold cell increments and then exposed the cells to PMA followed by DNA extraction and qPCR. When the ratio of living to dead cells was equivalent [log (ratio of dead to living cells) = 0], PMA-qPCR was accurate and there was no difference between viable numbers estimated by CFU enumeration and PMA-qPCR (**Figure 3**). Similarly, when the numbers of dead cells were 10-fold higher than living cells, PMA-qPCR was similarly in agreement with the culture-based assessment (**Figure 3**). However, PMA-qPCR on mixtures of 100-fold more dead cells than living cells [log (ratio of dead to living cells) = 2], this technique overestimated the number of viable cells in the mixture (**Figure 3**). Similarly, when only 10^6^ dead cells were exposed to PMA treatment, PMA-qPCR resulted in estimates of approximately 10^4^ viable cells. Hence, it is likely that the high number of viable *E. coli* ATCC 700728 estimated on lettuce and petri dishes by PMA-qPCR actually consisted of dead cells that lacked the capacity to recover to form viable and active populations.

### Detection of Dead *E. coli* and Degraded RNA by RT-qPCR

**FIGURE 4:**
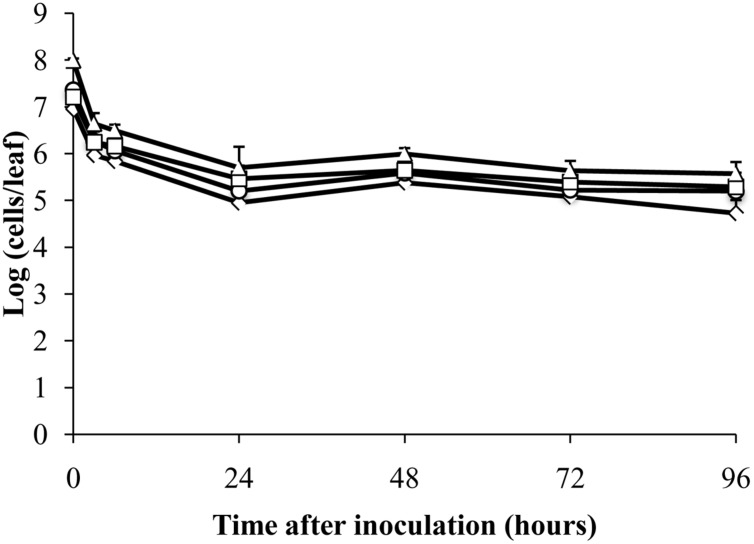
**Detection of isopropanol-treated dead *E. coli* O157:H7 on plants by RT-qPCR.** Cell numbers were estimated by RT-qPCR targeting *gapA* (♢), *eae* (□), *lpfA* (△), and *rfbE* (○) transcripts compared to standard curves constructed using cDNA from known quantities of viable, exponential-phase *E. coli* ATCC 700728 cells. Each point represents the mean ± standard deviation of three independent replicates.

To test whether mRNA could be detected in dead cells by RT-qPCR, *E. coli* ATCC 700728 was killed in 70% isopropanol and then inoculated onto either Romaine lettuce or sterile petri dishes. RNA in dead *E. coli* inoculated onto lettuce plants was highly degraded (ratio of 23S to 16S rRNA = 1.0) and then was further degraded after inoculation onto plants (ratio of 23S to 16S rRNA = 0.0). However, total cell numbers estimated by RT-qPCR only decreased about 100-fold compared with inoculum levels within the first 24 h (**Figure 4**). At subsequent time points, the estimated cell numbers remained relatively constant for all four target genes (**Figure 4**). Notably, cell numbers estimated using *gapA* transcripts as the target were significantly lower than those estimated for *rfbE* (Steel-Dwass, *P* = 0.0019) and *eae* (Steel-Dwass, *P* = 0.0002). The same trends were also found for *E. coli* ATCC 700728 inactivated with 70% isopropanol-inactivated and incubated on petri dishes (data not shown) and heat killed cells in suspension (**Supplementary Figure S3**).

**FIGURE 5:**
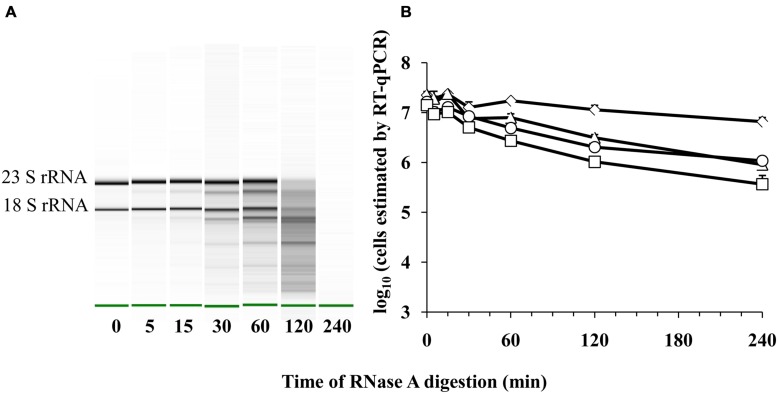
**Detection of *E. coli* O157:H7 RNA after RNase A digestion by RT-qPCR.** **(A)** Electrophoretic analysis of RNA integrity. The green band indicates the reference marker. **(B)** Cell number equivalents were estimated following RnaseA digestion by RT-qPCR targeting *gapA* (♢), *eae* (□), *lpfA* (△), and *rfbE* (○) transcripts compared to standard curves constructed using known quantities of viable, exponential-phase *E. coli* ATCC 700728 cells. Each point represents the mean ± standard deviation of three independent replicates.

Because cell death might result in the destruction of cellular RNases and reduce RNA turnover, we also measured whether a detection limit for *E. coli* transcripts could be reached upon increasing exposure of purified *E. coli* ATCC 700728 RNA to active RNases. Exposure to RNase A between 0 and 240 min resulted in significant reductions in intact ribosomal RNA and presumably mRNA transcripts (**Figure 5A**). By 120 min of exposure to RNase A, the RNA was degraded to the extent that 16S and 23S RNA were no longer detected by capillary electrophoresis (**Figure 5A**). However, there was only a 0.3 log_10_ reduction in cell number equivalents according to RT-qPCR on *gapA* transcripts and only a 10-fold reduction when *eae, lpfA*, and *rfbE* transcripts were targeted for detection (**Figure 5B**).

## Discussion

Because bacterial foodborne pathogens encounter a variety of environmental stresses on plants in the field and post-harvest during processing and packaging, it is likely that only a small fraction of cells are able to survive and cause human infection after a contamination event. Therefore, ideal detection methods should only measure viable pathogen numbers (Law et al., 2014). Our findings show that bacterial detection and enumeration on lettuce according to RT-qPCR and PMA-qPCR are not accurate and can detect dead *E. coli* O157:H7. Hence, applications of these techniques for foodborne pathogens should be used with caution when applied for viable bacteria cell detection and enumeration on fresh produce.

Measuring bacterial gene transcripts by RT-(quantitative) PCR has been used as an indicator of viability for a variety of foodborne pathogens in different foods (e.g., Klein and Juneja, 1997; McIngvale et al., 2002; Miller et al., 2010; Zhou et al., 2014). However, transcript detection is not always correlated with the presence of living and intact cells (Xiao et al., 2012). There are a variety of possible reasons for these different findings. To this regard, longer RNAs are more likely to undergo additional hydrolysis and become undetectable in subsequent cDNA synthesis and qPCR steps. This difference could explain the validation of transcript-detection by standard RT-PCR for longer (>200 nucleotides) products (Sheridan et al., 1998; Szabo and Mackey, 1999) as opposed to the shorter products typically produced in qPCR assays. Similarly, PMA-qPCR is also more accurate when used to measure longer DNA targets (Moyne et al., 2013). This is possibly consistent with the PMA-qPCR assessments for the four different gene targets measured here. Notably, however, even though there was a 60 bp range in transcript length among the *E. coli* mRNAs used for RT-qPCR in this study, these size differences were not correlated with estimates of cell viability.

Another possibility for the differences in RT-qPCR viability estimates between studies is the inherent stability of the transcripts tested. For example, 16S rRNA was previously found to be more stable than cellular mRNAs (Sheridan et al., 1998). However, remarkably, we found that the detection of different protein-encoding, *E. coli* O157:H7 ATCC 700728 transcripts by RT-qPCR was not broadly diminished with extended exposure to ribonuclease. Only a 0.6–1.6 log_10_ decrease in cell number equivalents was observed according to RT-qPCR on the digested RNA. Although after ribonuclease treatment there were increased levels of *gapA* mRNA (and hence *E. coli* cell number equivalents enumerated) compared to the three other transcripts tested, this difference was not consistent with gene-specific, transcript-based measurements of *E. coli* ATCC 700728 on plants or on petri dishes. These findings suggest that ribonucleases targeting single-stranded RNAs, such as the RnaseA used here, are not fully effective at digesting mRNA transcripts to lengths smaller than necessary for qPCR detection.

Another factor that can alter RT-qPCR measurements of bacterial viability is the lethal treatment used for cell inactivation. Prior studies on the development of this method used heat or exposure to solvents (ethanol) to test the capacity of RT-qPCR to measure viable cell numbers. Transcripts were found to be more stable after exposure of bacteria to ethanol as opposed to heat (Sheridan et al., 1998). Herein we found that exposure of *E. coli* O157:H7 ATCC 700728 to solvent (isopropanol) or heat yielded similarly erroneous estimates of viability with RT-qPCR. We also extended this comparison to *E. coli* ATCC 700728 exposed to low %RH desiccation stress on a sterile plastic surface and reached similar results. The only notable exception for *E. coli* O157:H7 ATCC 700728 survival on lettuce was that there was a 100-fold reduction in estimated viable cell numbers by RT-qPCR starting 48 h after application of that strain onto the plants. This reduction might have been due to predation on the dead *E. coli* cells remaining on the lettuce or other factors that remain to be determined.

Examination of *E. coli* O157:H7 under low %RH conditions in the laboratory is comparable to the conditions that those foodborne pathogens are exposed to on fresh produce in the field. The phyllosphere, or areal surfaces of plants, is regarded to be a harsh environment for microorganisms because it contains limited quantities of nutrients available for cell growth and is subject to rapid changes in temperature, moisture, ultraviolet radiation (Lindow and Brandl, 2003) that likely occur over diurnal and seasonal scales (Williams et al., 2013). Among these stresses, exposure to low %RH is a major determinant of *E. coli* survival. On lettuce plants maintained at near 100% RH, *E. coli* cell numbers increase rapidly and reach population sizes of 10^9^ CFU/g (Cooley et al., 2003). By comparison, inoculation onto plants maintained at low (60%) RH results in rapid declines in culturable cell numbers to levels similar to those detected for *E. coli* O157:H7 on field lettuce in the Salinas Valley, CA (Moyne et al., 2013). In the present study, the majority of culturable *E. coli* O157:H7 ATCC 700728 declined within 48 h after inoculation on Romaine lettuce maintained at low (30%) RH. A similar reduction in viable cell numbers was observed according to PMA-qPCR. Although the exposure of the pathogen to desiccation conditions on the abiotic (petri dish) surface resulted in more rapid losses in culturable cell numbers, exposure to both biotic (lettuce) and abiotic (petri dish) surfaces yielded similarly low levels of surviving *E. coli* cells. In general, a lack of available water results in desiccation stress and induction of a variety of cellular responses including a reduction in capsular layers, increase in salt concentration, reduction in membrane fluidity (Potts, 1994). These changes might also reduce the availability of water required for RNase activity (Malin et al., 1991), and therefore could have contributed to the high estimates of viable cell numbers by RT-qPCR.

To the best of our knowledge, this was the first study to apply RT-qPCR to detect and enumerate viable foodborne pathogen cell numbers on plant surfaces. The qPCR methods developed and applied here, although not high-throughput, are useful because they can be performed more rapidly than culture-based assessments. Of particular interest was the application of RT-qPCR to enumerate viable *E. coli* O157:H7 cells. Although this approach could be useful for the general detection of *E. coli* O157:H7, we have unequivocally shown the lack of association between *E. coli* O157:H7 mRNA transcript abundance and cell viability. We demonstrated that transcripts of sufficient length for detection by qRT-PCR were present in *E. coli* O157:H7 cells long after death and even after exposure of purified cellular RNA to exogenous ribonuclease. This knowledge is crucial when developing RT-PCR or other RNA related detection methods such as those in development for next-generation bio-sensors (Law et al., 2014). Moreover, RNA stability should be taken into account during experiment design and data interpretation, especially under low-moisture environments such as field-grown plants.

## Author Contributions

Conceptualization, MM; Methodology, WJ, A-LM and MM; Investigation, WJ and A-LM; Formal Analysis, WJ; Writing, Reviewing and Editing, WJ, A-LM and MM; Funding Acquisition, MM; Supervision, MM.

## Conflict of Interest Statement

The authors declare that the research was conducted in the absence of any commercial or financial relationships that could be construed as a potential conflict of interest.
