# Supplementary material for: RNA-Based Detection Does not Accurately Enumerate Living Escherichia coli O157:H7 Cells on Plants
Source: Front Microbiol. 2016 Feb 26;7:223. doi: 10.3389/fmicb.2016.00223 (PMC4767924; doi:10.3389/fmicb.2016.00223)
Supplement: Supplementary file 1 [file Data_Sheet_1.PDF]

Supplementary Material

RNA-based dectection does not accurately enumerate living *Escherichia coli* O157:H7 cells on plants

Wenting Ju, Anne-laure Moyne, Maria L. Marco*

*** Correspondence:** Corresponding Author: mmarco@ucdavis.edu

Supplementary Figures

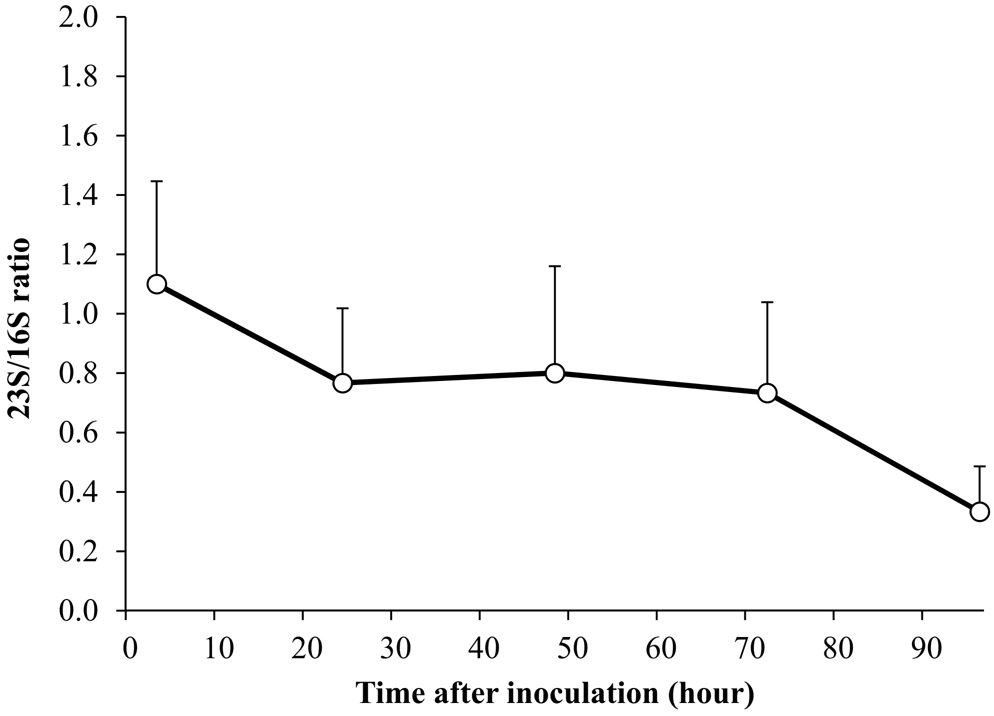

Supplementary Figure 1. Ratio of bacterial 23S to 16S rRNA after inoculation of *E. coli* O157:H7 ATCC 700728 on Romaine lettuce in the growth chamber at 30% RH. Each sampling point represents the mean ± stdev of three independent replicates.

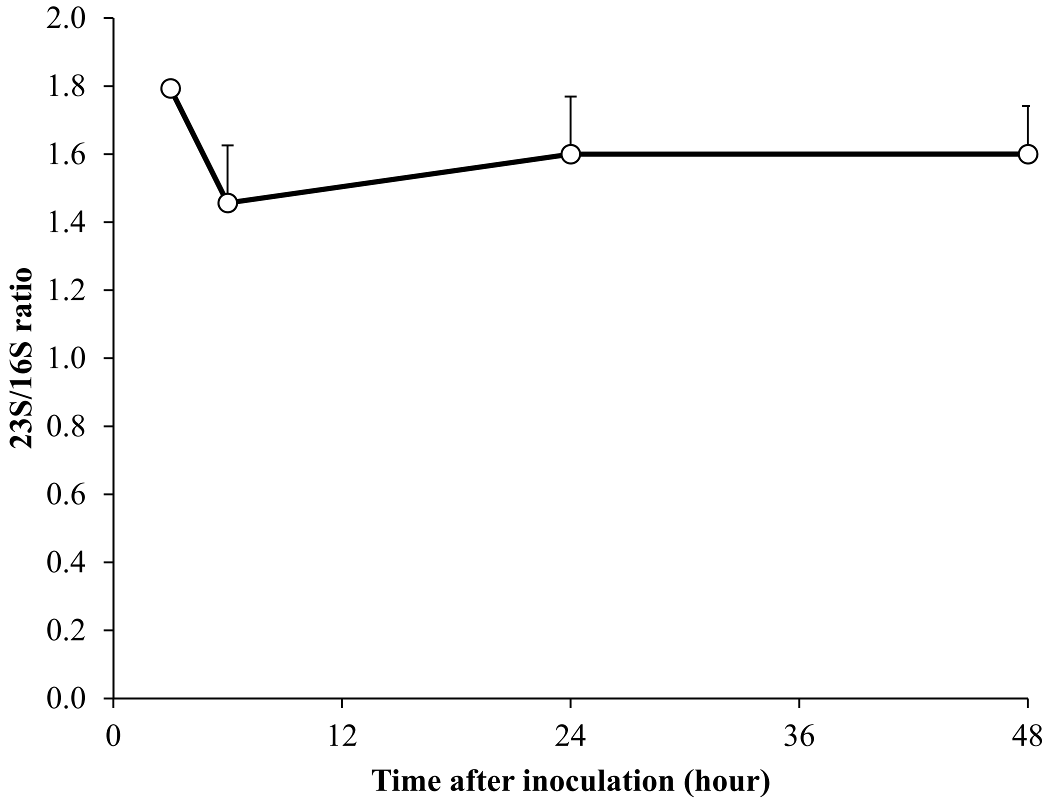

Supplementary Figure 2. Ratio of *E. coli* O157:H7 ATCC 700728 23S to 16S rRNA after inoculation onto a sterile petri dish. Each sampling point represents the mean ± stdev of three independent replicates.

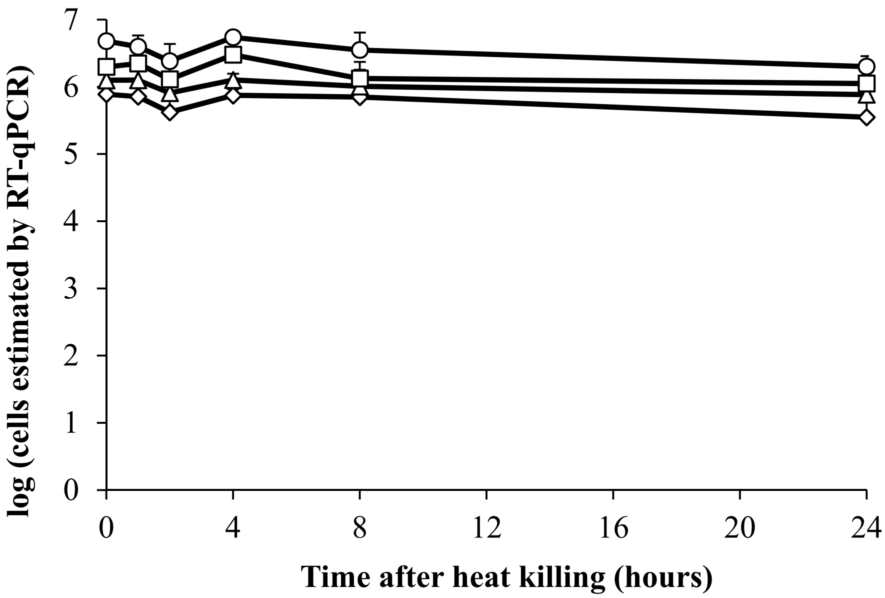

Supplementary Figure 3. Detection of heat-killed *E. coli* O157:H7 cells in PBS by RT-qPCR. Cell numbers were estimated by RT-qPCR targeting *gapA* (◇), *eae* (□), *lpfA* (△) and *rfbE* (○) transcripts compared to standard curves constructed using known quantities of *E. coli* ATCC 700728 cells. Each point represents the mean ± stdev of three independent replicates.
